# Enhanced Yield of Methyl Ethyl Ketone through Levulinic Acid Decarboxylation in the AgNO_3_/K_2_S_2_O_8_ System: Mechanistic Insights and Characterization of Metallic Species

**DOI:** 10.3390/molecules29204822

**Published:** 2024-10-11

**Authors:** Nydia I. Guzmán Barrera, Jérôme Peydecastaing, Jérôme Esvan, Joël Albet, Carlos Vaca-Garcia, Philippe Behra, Emeline Vedrenne, Sophie Thiébaud-Roux

**Affiliations:** 1 Laboratoire de Chimie Agro-Industrielle (LCA), Université de Toulouse, INRAE, Toulouse INP, 31030 Toulouse, France; nydiaiguzman@gmail.com (N.I.G.B.); jerome.peydecastaing@toulouse-inp.fr (J.P.); joel.albet@toulouse-inp.fr (J.A.); carlos.vacagarcia@toulouse-inp.fr (C.V.-G.); philippe.behra@toulouse-inp.fr (P.B.); emeline.vedrenne@ensiacet.fr (E.V.); 2 CIRIMAT, Université de Toulouse, Toulouse INP, CNRS, 31030 Toulouse, France; jerome.esvan@ensiacet.fr

**Keywords:** decarboxylation mechanism, methyl ethyl ketone synthesis, thermodynamic calculations, solid intermediate characterization, levulinic acid reactivity

## Abstract

Methyl ethyl ketone (MEK) is among the most extensively utilized solvents in various industrial applications. In this study, we present a highly efficient synthesis route for MEK via the decarboxylation of biomass-derived levulinic acid, using potassium persulfate (K_2_S_2_O_8_) and silver nitrate (AgNO_3_) as key reagents. The specific roles of AgNO_3_ and K_2_S_2_O_8_ were thoroughly investigated. Additional silver species, such as Ag_2_O and AgO, were also detected during the reaction. X-ray photoelectron spectroscopy (XPS) and X-ray diffraction (XRD) analyses provided evidence of the evolution of solid phases throughout the reaction. Based on these findings, we propose a radical decarboxylation mechanism initiated by the generation of sulfate radicals (SO_4_•⁻) through the catalytic breakdown of K_2_S_2_O_8_ by AgNO_3_. This mechanistic understanding, combined with a parametric study, enabled us to achieve an unprecedented level of levulinic acid conversion (97.9%) and MEK yield (86.6%) with this system, surpassing all previously reported results in the literature.

## 1. Introduction

Methyl ethyl ketone (MEK) is widely utilized in the paint and surface coating industries, primarily as a low-boiling-point solvent for nitrocellulose, acrylic, and vinyl compounds. It is also frequently employed in professional paint shops and by painters for thinning acrylics and lacquers [1,2].

In the context of a growing bioeconomy, the use of biomass feedstocks has become increasingly attractive as a means to reduce carbon footprints and decrease reliance on fossil resources. Therefore, research into the synthesis of MEK from renewable raw materials, such as levulinic acid (LA), is particularly relevant, especially when utilizing environmentally sustainable processes.

Levulinic acid has been identified by the National Renewable Energy Laboratory (NREL) as one of the most valuable chemicals that can be produced from sugars or lignocellulosic biomass through acid dehydration [3]. It is an abundant chemical building block, with reported demand reaching 2606.2 tons in 2013 [4]. LA serves as a precursor to a wide range of valuable compounds, including tetrahydrofuran (THF), 2-methyltetrahydrofuran (2-MeTHF), levulinate esters, γ-valerolactone (GVL), α-angelica lactone, 1,4-pentanediol (PDO), acrylic acid, β-acetyl acrylic acid, and α-aminolevulinic acid [3,5].

The decarboxylation of heteroaromatic carboxylic acids [6,7,8,9], particularly with the use of high temperatures and Brønsted-Lewis acid catalysts such as SiO_2_-Al_2_O_3_ and γ-Al_2_O_3_ [10], has been extensively studied, whereas the decarboxylation of aliphatic carboxylic acids like LA remains less explored. Hydroxyl radicals (•OH) have been reported in the decarboxylation of aliphatic organic acids, such as 2-methylalanine and amino acids [11,12,13,14,15]. Furthermore, metal oxidants including Pb(IV), Co(III), Mn(III), Fe(II), Cu(II), and Ag (I) have also been examined [16,17,18,19,20,21].

Only a few studies have specifically addressed the decarboxylation of LA. Chum et al. reported the photoelectrochemical decarboxylation of LA using n-TiO_2_ as a semiconductor, but the MEK yield was very low (0.0025–0.01%) [22]. In addition to MEK, products such as methanol, ethanol, propionic acid, acetic acid, acetone, acetaldehyde, ethyl acetate, methane, and ethane were also observed. More recently, Gong et al. described LA decarboxylation using cupric oxide as a catalyst under harsh conditions (300 °C), achieving a MEK yield of 67.5%, alongside acetone and acetic acid production [23]. Gong et al. also investigated LA decarboxylation using AgNO_3_/K_2_S_2_O_8_ as co-reagents at 160 °C for 30 min in an aqueous NaOH/KH_2_PO_4_ solution at pH 5, obtaining a moderate MEK yield of 44.2% under these milder conditions [24]. Mehrer et al. explored the decarboxylation of LA using an engineered *E. coli* strain, yielding 76% MEK [25]. Additionally, LA decarboxylation as a side reaction during LA hydrodeoxygenation with sulfided NiMo/Al_2_O_3_ catalyst was reported by Grilc and Likozar [26], while low yields of MEK (6%) were observed during LA hydroconversion with Ni/SiO_2_ as a catalyst [27].

Decarboxylation reactions involving the Ag(I)/K_2_S_2_O_8_ system have garnered significant interest from researchers [28,29,30,31,32,33,34]. It has been proposed that the decarboxylation mechanism in this system involves the oxidation of Ag(I) to Ag(II) by S_2_O_8_^2^⁻, with Ag(II) subsequently promoting the formation of carboxyl radicals, leading to decarboxylation and the formation of the corresponding alkyl radicals [35].

The primary goal of this study was to optimize the decarboxylation of LA for MEK synthesis using the AgNO_3_/K_2_S_2_O_8_ system under milder conditions and to elucidate the reaction mechanism. We first investigated the speciation of silver and its interaction with K_2_S_2_O_8_. Through the optimization of experimental conditions, we achieved an exceptional MEK yield of 86.6%. Finally, XPS and XRD analyses were performed to characterize the solid phases involved, providing strong support for the proposed reaction mechanism.

## 2. Results and Discussion

The decarboxylation of levulinic acid (LA) was studied in an aqueous phosphate solution, following the methodology previously described by Gong et al. [23,24]. The experimental setup involved multiphase systems composed of solid Ag salts, an aqueous solution containing LA and K_2_S_2_O_8_, and a gaseous phase corresponding to the vapor pressure of the reactants and products.

### 2.1. Influence of K_2_S_2_O_8_ and AgNO_3_

To clarify the individual roles of K_2_S_2_O_8_ and AgNO_3_ in LA decarboxylation, reactions were conducted at 100 °C without one of these components. When silver nitrate was added alone, no LA decarboxylation was observed after 30 min of reaction (Table 1, entry #1). However, when K_2_S_2_O_8_ was used without AgNO_3_, the reaction proceeded to varying degrees depending on the conditions. Using 0.25 equivalents of K_2_S_2_O_8_ yielded trace amounts of methyl ethyl ketone (MEK) (2.6%) and acetic acid (AcOH) (1%) (Table 1, entry #2). Increasing the persulfate concentration raised LA conversion from 3.6% to 18.8% (Table 1, entries #2–#5). The thermal decomposition of persulfate to generate sulfate radicals (SO_4_•–) in aqueous solutions, as previously reported [30,36,37,38,39,40,41,42,43], likely explains the decarboxylation observed without AgNO_3_. However, at higher K_2_S_2_O_8_ concentrations (1–2 equivalents), LA conversion increased while MEK yield decreased (to 4.3%), with a corresponding increase in acetic acid production (14.5%) (Table 1, entries #4 and #5).

In the absence of AgNO_3_, the decarboxylation of LA by thermal persulfate decomposition at low pH (pH < 3; 100 °C) can be explained by the mechanism described in Equations (1)–(4).

(1) $$S_{2}{O_{8}}^{2-}\;\to \;2\;S{O_{4}}^{-}•$$

(2) $$CH_{3}\mathrm{COC}H_{2}CH_{2}\mathrm{COOH}+S{O_{4}}^{-}•\;\to \;CH_{3}\mathrm{COC}H_{2}CH_{2}\mathrm{COO}\text{•}+\mathrm{HS}{O_{4}}^{-}$$

(3) $$CH_{3}\mathrm{COC}H_{2}CH_{2}\mathrm{COO}\text{•}+H_{2}O\;\to \;CH_{3}\mathrm{COC}H_{2}CH_{3}+CO_{2}+\mathrm{OH}\text{•}$$

(4) $$2\;\mathrm{OH}•\;\to {\;H}_{2}O+\frac{1}{2}{\;O}_{2}$$

**Figure 1:**
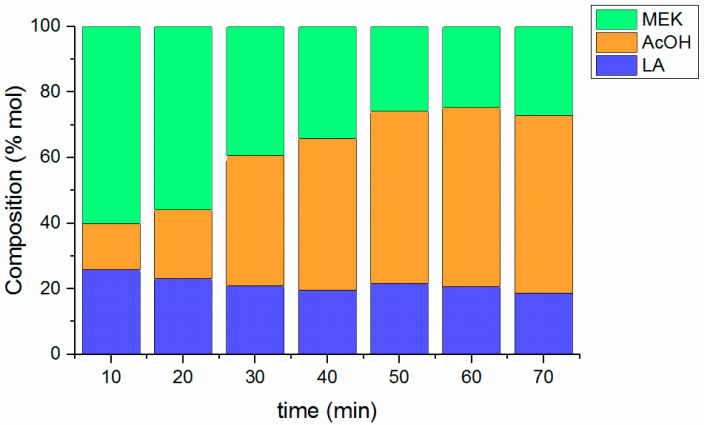
Decarboxylation of LA with 1 eq. of K_2_S_2_O_8_ in the absence of AgNO_3_. Experimental conditions: K_2_HPO_4_/KH_2_PO_4_ (0.1/0.1 M), 100 °C.

The role of K_2_S_2_O_8_ in MEK production was further examined by investigating the reaction kinetics with 1 equivalent of K_2_S_2_O_8_ (Table 1). The MEK yield was highest in the early stages of the reaction but began to decline as acetic acid production increased (Figure 1). This behavior was explored by subjecting MEK to the same reaction conditions in the presence of potassium persulfate. A 10% conversion of MEK into acetic acid was observed after 30 min at 100 °C (see Section 2.3, Scheme 2). These results indicate that during LA decarboxylation, MEK is partially oxidized to acetic acid, explaining the drop in MEK yield and the rise in acetic acid after 20 min of reaction. At 25 °C, MEK was also converted to acetic acid but in smaller amounts, suggesting that temperature plays a crucial role in MEK oxidation to AcOH. 

### 2.2. Role of Ag Salts

The decomposition of K_2_S_2_O_8_ is known to be catalyzed by transition metals, including silver species [17,35,44]. To investigate the influence of different silver salts on LA decarboxylation, reactions were performed using a 1:1 ratio of Ag salt to K_2_S_2_O_8_ at 100 °C. The presence of AgNO_3_ significantly enhanced LA conversion (from 14% to 46.8%) and MEK yield (from 6.8% to 32%) (Table 1, entry #4 vs. Table 2, entry #1). With AgCl, MEK yield was slightly lower (25.2%) (Table 2, entry #2). In contrast, when Ag_2_O or AgO was used with persulfate, MEK yields were comparable to AgNO_3_, but acetic acid production increased substantially (Table 2, entries #3 and #4). These results suggest that silver species catalyze K_2_S_2_O_8_ decomposition, enhancing LA decarboxylation.

The decarboxylation mechanism involving Ag(I) and K_2_S_2_O_8_, first proposed by Anderson and Kochi in 1970 [35] and later by other authors [24,32,45,46], suggests a catalytic cycle in which Ag(I) is oxidized to Ag(II) by persulfate or sulfate radicals. Ag(II) then promotes decarboxylation, regenerating Ag(I). A reaction with 1 equivalent of AgO (Ag(II)) without K_2_S_2_O_8_ resulted in no LA conversion (Table 2, entry #5), confirming that K_2_S_2_O_8_ is essential for LA decarboxylation. This points to a radical mechanism initiated by sulfate radicals (SO_4_•–) from K_2_S_2_O_8_ decomposition. Our results are consistent with those reported by Seipled et al., who found that no arylation of various heterocycles occurred with arylboronic acid in the presence of Ag(II) as the sole oxidant [31]. 

### 2.3. Role of K_2_S_2_O_8_ in the Presence of AgNO_3_

**Scheme 1:**
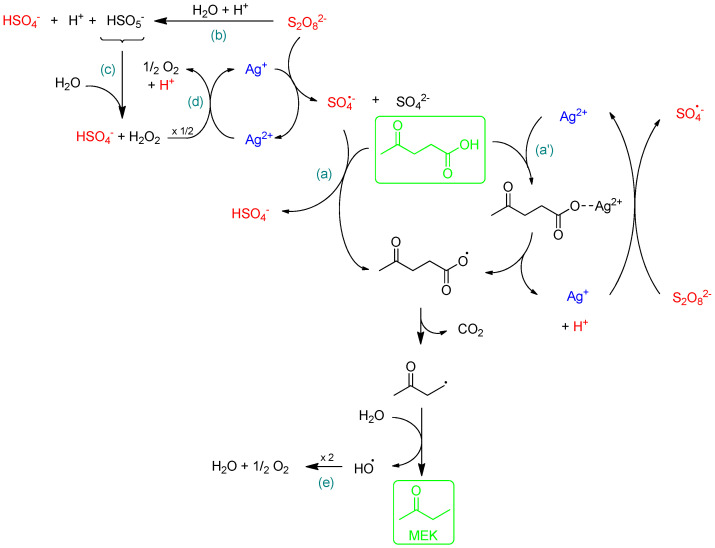
Proposed mechanisms.

To verify that levulinic acid (LA) decarboxylation proceeds via a radical mechanism, initiated by the decomposition of K_2_S_2_O_8_ into sulfate radicals (SO_4_•⁻), a reaction was performed with the radical scavenger TEMPO ((2,2,6,6-tetramethylpiperidin-1-yl)oxyl). The reaction conditions were the same as those used for AgNO_3_/K_2_S_2_O_8_ (1/1 equivalent) with the addition of 1 or 2 equivalents of TEMPO. The complete suppression of LA conversion confirmed the radical nature of the reaction, specifically that the persulfate ions break down into SO_4_•⁻, which are responsible for LA decarboxylation [Reaction (a), Scheme 1]. Additionally, the pH of the solution decreased during the reaction, attributed to the formation of bisulfate ions (HSO_4_⁻), a weak acid (pKa_2_ = 1.9 at 25 °C and 2.98 at 99 °C) [Equation (5)].

(5) $$H_{2}S{O_{4}}^{-}+H_{2}O\;↔\;H_{3}O^{+}+\mathrm{HS}{O_{4}}^{2-}$$

**Scheme 2:**
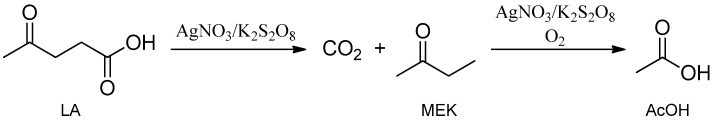
AcOH production via MEK oxidation.

Under such acidic conditions, S_2_O_8_^2^⁻ can further decompose into sulfuric acid and Caro’s acid (H_2_SO_5_), [Reaction (b), Scheme 2] [37], contributing to the observed pH decrease. The formation of hydrogen peroxide (H_2_O_2_) [Reaction (c)] and its subsequent reduction to oxygen (O_2_) leads to the regeneration of Ag(I) [Reaction (d), Scheme 2]. Additionally, a reaction between levulinic acid and Ag(II) may occur [Reaction (a’), Scheme 2], producing the same radical intermediates along with Ag(I) and H⁺, which further decreases the solution pH. 

Given that the AgNO_3_/K_2_S_2_O_8_ system provided the highest MEK yields and the lowest acetic acid (AcOH) production (Table 2, entry #1), this combination was selected for further parametric study to gain deeper insight into the reaction mechanism.

### 2.4. Influence of AgNO_3_/K_2_S_2_O_8_ Ratio

A series of reactions were conducted with varying AgNO_3_/K_2_S_2_O_8_ ratios. Lower LA conversions and MEK yields were observed with smaller amounts of AgNO_3_ and K_2_S_2_O_8_ (Table 3, entries #1 and #2). As observed in the absence of AgNO_3_ (Table 1, entry #5), increasing the amount of K_2_S_2_O_8_ to 2 equivalents led to increased AcOH production but did not enhance the MEK yield (Table 3, entry #4). 

To confirm whether acetic acid forms as a result of MEK oxidation, a reaction was performed using MEK as the starting material in the presence of 1 equivalent each of AgNO_3_ and K_2_S_2_O_8_ at 100 °C. MEK conversion into AcOH was observed to be 10% and 17% after 30 and 60 min, respectively. This supports the hypothesis that during LA decarboxylation under acidic conditions, MEK can undergo subsequent oxidation to AcOH (Scheme 2). These findings align with those of Hobbs et al. [47], who demonstrated that MEK is oxidized to AcOH in the presence of O_2_ and a metal catalyst. In this system, O_2_ is generated during the reduction of Ag(II) to Ag(I) [Reaction (d), Scheme 1] and the termination reaction [Reaction (e), Scheme 1].

### 2.5. Influence of Temperature and Reaction Time

**Figure 2:**
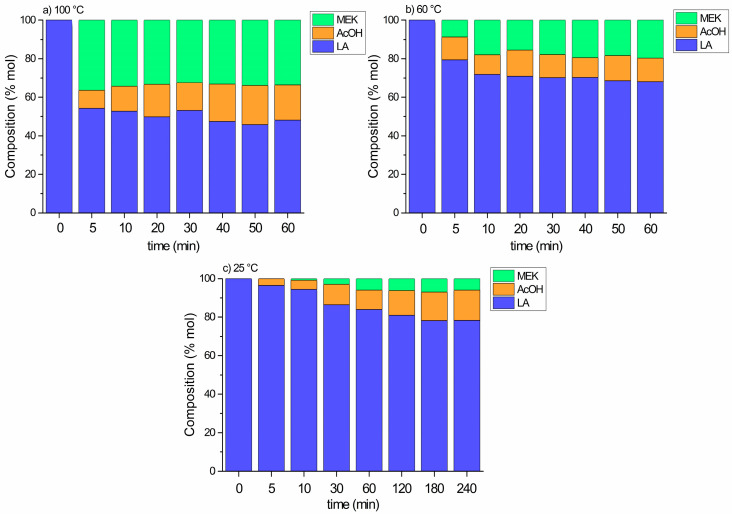
LA decarboxylation at different temperatures and reaction times (**a**) 100, (**b**) 60, and (**c**) 25 °C. Experimental conditions: AgNO_3_/K_2_S_2_O_8_ (1/1), KH_2_PO_4_/K_2_HPO_4_ (0.1/0.1 M).

A kinetic study of LA decarboxylation was conducted at 100 °C, 60 °C, and 25 °C to further understand and optimize MEK production (Figure 2). Reactions were carried out with 1 equivalent each of AgNO_3_ and K_2_S_2_O_8_. At 100 °C, MEK formation was faster than AcOH production, reaching a maximum yield after 5 min. At 60 °C, slightly more AcOH was produced, reaching 20% yield after 20 min, while MEK production was slower. At room temperature, more AcOH was produced than MEK (Figure 2c), suggesting that MEK oxidation to AcOH outpaces its production at lower temperatures.

Two phenomena may explain these observations: (i) at lower temperatures, O_2_ is more soluble in the aqueous medium, promoting MEK oxidation [47]; and (ii) at higher temperatures, such as 100 °C, some of the MEK may be in the gaseous phase. However, ProSim^®^ simulations of vapor–liquid equilibrium at 100 °C and atmospheric pressure indicated that less than 1% of MEK was in the gas phase, ruling out the second hypothesis. Therefore, MEK oxidation to AcOH appears to be linked to increased oxygen solubility at lower temperatures, while the increase in MEK production with temperature suggests that LA decarboxylation is an endothermic reaction, with the equilibrium shifting to favor MEK production at higher temperatures (Figure 2a). Similar behavior has been observed in the decarboxylation of malonic acid, butylmalonic acid, and 2-aminoisobutyric acid, where the reactions were also endothermic, with enthalpy changes (ΔH) ranging from 17.0 to 39.9 kcal/mol [48,49,50].

Given that low temperatures did not yield optimal results, reactions were conducted under identical conditions at 125 °C and 150 °C. However, no significant increase in MEK production was observed beyond 100 °C, with yields of 25% and 28% MEK at 125 °C and 150 °C, respectively.

### 2.6. Characterization of Solid Phases

**Figure 3:**
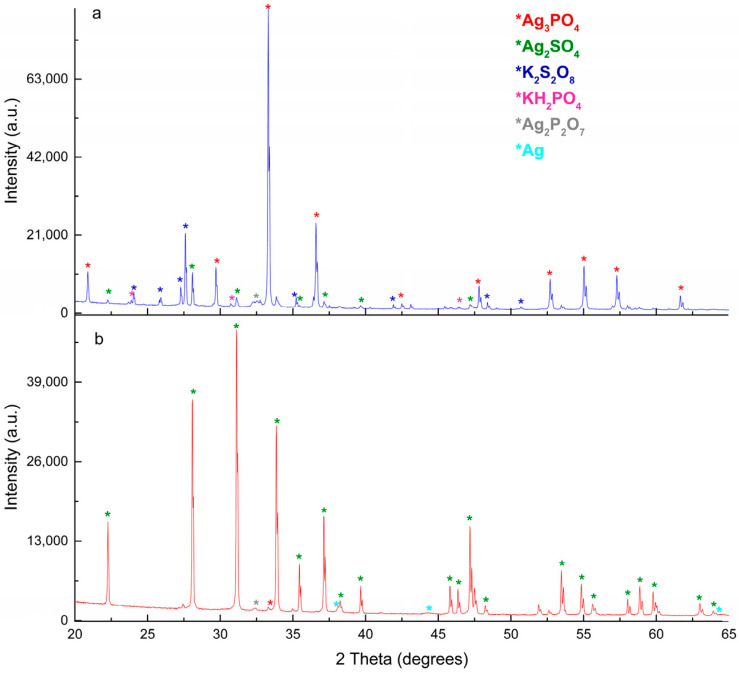
XRD patterns for the solid phase (**a**) before and (**b**) after the reaction. Experimental conditions: AgNO_3_/K_2_S_2_O_8_ (1/1), KH_2_PO_4_/K_2_HPO_4_ (0.2 M), 100 °C, 30 min.

X-ray diffraction (XRD) and X-ray photoelectron spectroscopy (XPS) were employed to examine the evolution of solid phases during the reaction, aiming to understand why MEK yields did not exceed 33%. A notable color change, from colorless to yellow, was observed upon the addition of AgNO_3_ to a solution of phosphates (KH_2_PO_4_/K_2_HPO_4_) containing levulinic acid (LA) and potassium persulfate (K_2_S_2_O_8_) at the start of the reaction, suggesting the formation of Ag_3_PO_4_. XRD analysis confirmed this, showing that the solid phases primarily consisted of Ag_3_PO_4_ with distinct diffraction peaks at 20.9°, 29.7°, 33.3°, 36.6°, 42.5°, 47.8°, 52.7°, 55.0°, 57.3°, 61.7°, 65.9°, 69.9°, and 71.9° (JCPDS No. 06-0505) [51]. Additionally, traces of K_2_S_2_O_8_ (major peaks at 24.0, 25.9, 27.3, 27.6, 29.3, 32.7, 35.2, and 36.4°; ICSD No. 16972) [52], Ag_2_SO_4_ (major peaks at 22.2, 31.1, 33.8, 37.2, 47.0, and 53.5°) [53], KH_2_PO_4_ (major peaks at 23.8, 30.7, and 46.5°; ICSD 201371) [54], and Ag_4_P_2_O_7_ (major peaks at 27.1, 32.3, and 32.5°) were also identified (Figure 3a).

Ag_3_PO_4_ likely forms from the reaction between AgNO_3_ and H_2_PO_4_⁻/HPO_4_^2^⁻, as represented by Equations (6) and (7).

(6) $$3\;\mathrm{AgN}O_{3}\left(s\right)+H_{2}P{O_{4}}^{-}\;↔{\;\mathrm{Ag}}_{3}PO_{4}\left(s\right)+3\;N{O_{3}}^{-}+2\;H^{+}$$

(7) $$3\;\mathrm{AgN}O_{3}\left(s\right)+\mathrm{HP}{O_{4}}^{2-}\;↔{\;\mathrm{Ag}}_{3}PO_{4}\left(s\right)+3\;N{O_{3}}^{-}+H^{+}$$

Subsequently, Ag_4_P_2_O_7_ may be produced according to Equations (8) and (9).

(8) $$2\;K_{2}\mathrm{HP}O_{4}\left(s\right)\;↔\;K_{4}P_{2}O_{7}\left(s\right)+H_{2}O$$

(9) $$4\;\mathrm{AgN}O_{3}\left(s\right)+K_{4}P_{2}O_{7}\left(s\right)\;↔{\;\mathrm{Ag}}_{4}P_{2}O_{7}\left(s\right)+4\;N{O_{3}}^{-}+4K^{+}$$

Finally, Ag_2_SO_4_ can form via the interaction of HSO_4_⁻ with AgNO_3_ (Equation (10)), with HSO_4_⁻ potentially generated by the decomposition of K_2_S_2_O_8_ in aqueous medium at low pH, initiated by AgNO_3_ or light (Equation (11)) [55,56,57,58].

(10) $$2\;K_{2}S_{2}O_{8}\left(s\right)+2\;H_{2}O\;↔\;4\;\mathrm{HS}{O_{4}}^{-}+4\;K^{+}+O_{2}\left(g\right)$$

(11) $$\mathrm{HS}{O_{4}}^{-}+2\;\mathrm{AgN}O_{3}\left(s\right)\;↔{\;\mathrm{Ag}}_{2}SO_{4}\left(s\right)+2\;N{O_{3}}^{-}+H^{+}$$

XRD patterns obtained at the end of the reaction (Figure 3b) revealed that the solid phases predominantly contained Ag_2_SO_4_, with traces of Ag_3_PO_4_, Ag_4_P_2_O_7_, Ag_3_O_4_, and elemental silver (Ag^0^). This suggests that Ag_3_PO_4_ was converted into Ag_2_SO_4_ during the reaction. The formation of Ag_2_SO_4_ likely occurred via the reaction between HSO_4_⁻ and silver phosphate, as indicated by Equation (12).

(12) $$2{\;\mathrm{Ag}}_{3}PO_{4}\left(s\right)+3\;\mathrm{HS}{O_{4}}^{-}+3\;H^{+}\;↔\;3\;{\mathrm{Ag}}_{2}SO_{4}\left(s\right)+2\;H_{3}PO_{4}$$

Additionally, diffraction peaks for Ag^0^ were observed at 38.6° (111), 44.3° (200), 64.4° (220), and 77.3° (311) [59], implying that elemental silver may form during the reaction. Previous studies by Deb et al. using XPS analysis showed Ag^0^ formation during the oxidative trifluoromethylation of alkenes with AgNO_3_/K_2_S_2_O_8_, suggesting a catalytic cycle involving Ag(I)-Ag(0) [60]. Hatamura et al. also reported the reduction of Ag(I) to Ag(0) during the decarboxylation of Ag(I) β-ketocarboxylates [61].

XRD patterns revealed weak peaks at 27.7°, 33.5°, 35.8°, and 39.7° in the solid phase at the end of LA decarboxylation, which were attributed to Ag_3_O_4_ (ICSD No. 59225). However, the presence of Ag_3_O_4_ was deemed unlikely as this compound decomposes under X-ray irradiation at room temperature [62]. Ag_3_O_4_ is a binary oxide Ag^2+^(Ag^3+^)_2_O_4_ [63], and its detection may be related to the presence of other oxide species.

**Figure 4:**
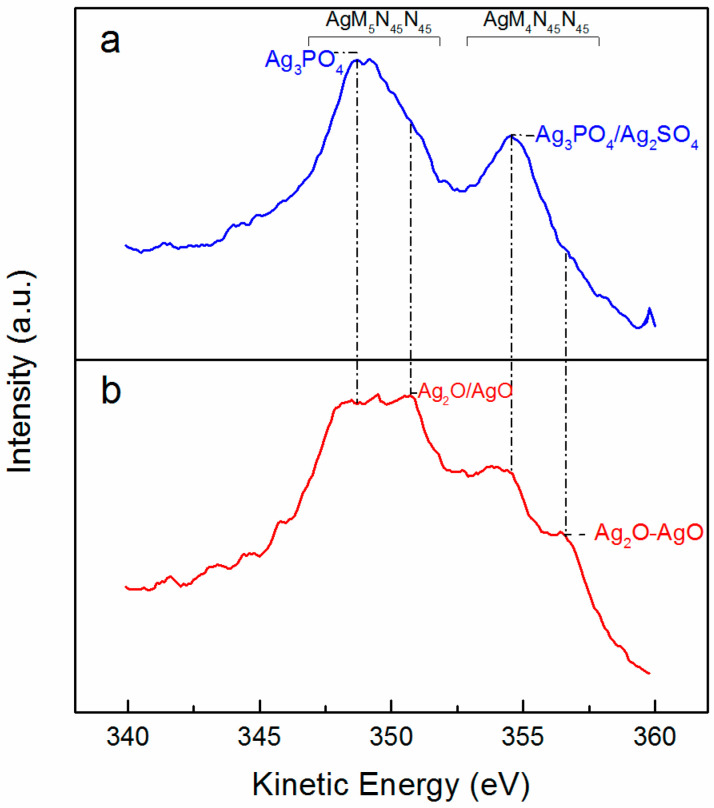
AgMNN Auger signals of the solid phase (**a**) before and (**b**) after levulinic acid decarboxylation.

XPS surface analyses were conducted to gain further insight into LA decarboxylation. The Ag 3d_5_/_2_ XPS spectrum at the beginning of the reaction showed a binding energy (BE) of 367.9 eV with a full width at half maximum (FWHM) of 1.17 eV (for Ag metal reference, FWHM < 0.8 eV), indicating a single silver oxidation state [64]. After the reaction, a slight shift to higher BE (368.05 eV) was observed, along with an increased FWHM of 1.46 eV, suggesting the presence of multiple silver species. The small BE shift made it difficult to determine the exact silver species involved, but Auger electron analysis revealed Ag oxide species. In the Auger spectra before and after the reaction (Figure 4), signals corresponding to AgM_5_N_45_N_45_ and AgM_4_N_45_N_45_ peaks were observed at 348.6 eV and 354.5 eV, which were attributed to Ag_3_PO_4_ [65]. A peak at 354.5 eV was also assigned to Ag_2_SO_4_ [66]. Furthermore, the presence of sulfates (S 2p_3/2_ at 169.24 eV) and phosphates (P 2p_3/2_ at 133.17 eV) was confirmed by XPS, corroborating the XRD results. After the reaction, additional Auger signals at 350.7 eV and 356.6 eV were attributed to Ag_2_O and AgO [67,68,69].

While Ag_3_O_4_ was identified in XRD patterns, neither Ag_2_O nor AgO were detected. These species may be present in amorphous forms on the solid surface, as indicated by XPS analyses. The mixed-valence nature of Ag_3_O_4_ (Ag^2^⁺(Ag^3^⁺)_2_O_4_) may have interfered with the identification of Ag_2_O and AgO in XRD measurements.

Solid-phase analyses primarily revealed the presence of Ag(I) in various forms, such as Ag_3_PO_4_, Ag_2_SO_4_, and Ag_4_P_2_O_7_, resulting from its interaction with the solution. Only trace amounts of Ag(II) and metallic Ag(0) were detected. The persistence of Ag(I) species throughout the reaction does not fully account for the observed limitations in LA decarboxylation. This may be attributed to the acidification of the reaction medium, which likely inhibits the generation of sulfate radicals (SO_4_•⁻) and instead promotes the formation of Caro’s acid, a competing reaction pathway [Scheme 1, Reaction (b) vs. Reaction (a)].

### 2.7. Influence of pH Variation

As described earlier, the pH of the reaction solution decreased during the reaction. The pH dropped from 5 to 2 or 1 as the concentration of K_2_S_2_O_8_ increased, with neither the K_2_HPO_4_/KH_2_PO_4_ buffer solution nor other tested acid-base pairs able to stabilize the pH. A drop in LA conversion and MEK yield was observed when NaOH was used as the base (Table 4, entries #1 and #8). However, similar conversion rates and yields were obtained with K_2_HPO_4_/KH_2_PO_4_ and KH_2_PO_4_ solutions (Table 4, entries #2–#4 and #6). MEK yields slightly improved with K_2_HPO_4_ and Na_2_HPO_4_/NaH_2_PO_4_ solutions (Table 4, entries #5 and #7).

To counteract the decreasing pH, NaOH was added to adjust the solution pH to 5 after 30 min of reaction. When the reaction was allowed to proceed for an additional 30 min under these conditions, there was no further increase in LA conversion or MEK yield (Table 5, entries #1 and #2). However, when both NaOH and an additional equivalent of persulfate were introduced after 30 min, LA conversion increased significantly to 99%, and an excellent MEK yield of 87% was achieved, underscoring the importance of maintaining pH and persulfate concentration (Table 5, entry #3). These results indicate that the drop in pH during the reaction limits MEK production. When persulfate and silver nitrate were added without pH adjustment, a modest increase in MEK yield from 31% to 38% was observed (Table 5, entry #5). However, in both cases, AcOH production increased. Thus, MEK production appears to be pH-dependent.

XRD and XPS analyses of the solid phase were conducted following the addition of NaOH to the reaction medium and after the second reaction cycle to investigate the underlying causes for the observed increase in levulinic acid (LA) conversion and corresponding enhancement in MEK production after pH adjustment.

**Figure 5:**
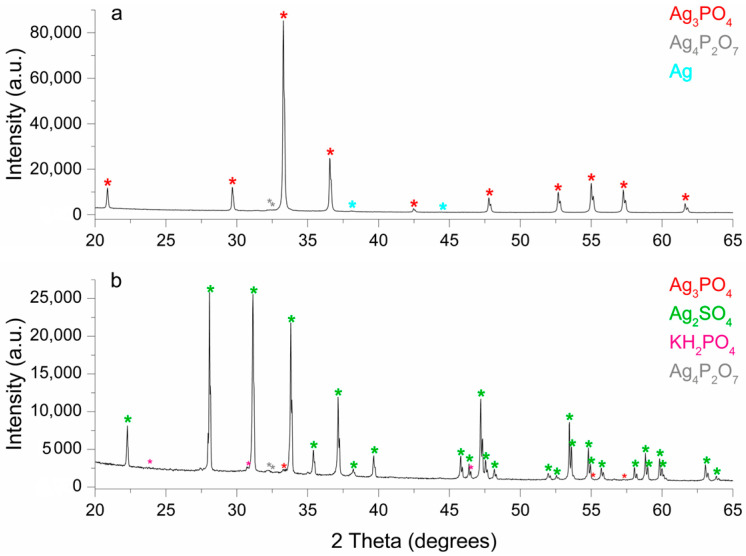
XRD patterns for the solid phase: (**a**) after M1 neutralization, (**b**) after the second cycle of the reaction, with the addition of K_2_S_2_O_8_. Experimental conditions: AgNO_3_/K_2_S_2_O_8_ (1/1), KH_2_PO_4_/K_2_HPO_4_ (0.1/0.1 M), 100 °C.

XRD patterns revealed that after pH adjustment with NaOH, the primary crystalline phase present was Ag_3_PO_4_, with minor amounts of Ag_4_P_2_O_7_ and metallic silver (Ag^0^) also detected (Figure 5a). The solid phase composition of the adjusted medium closely resembled that observed in the XRD patterns at the start of the first reaction cycle (Figure 3a). These findings suggest that the reaction medium can be regenerated by NaOH addition, which accounts for the improved LA conversion in the second reaction cycle.

The XRD patterns following the second reaction cycle are shown in Figure 5b. The conversion of Ag_3_PO_4_ to Ag_2_SO_4_ was observed, consistent with the results from the first reaction cycle (Figure 3b). Ag_2_SO_4_ was the dominant species present, along with residual traces of Ag_3_PO_4_, KH_2_PO_4_, and Ag_4_P_2_O_7_.

**Figure 6:**
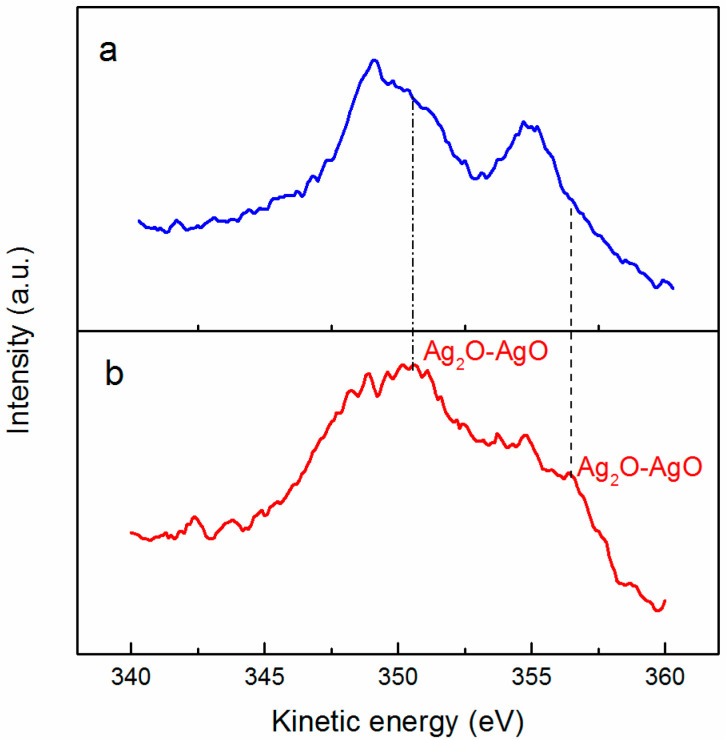
AgMNN Auger signals for the solid phase: (**a**) after M1 neutralization, (**b**) after the second cycle of reaction. Experimental conditions: AgNO_3_/K_2_S_2_O_8_ (1/1), KH_2_PO_4_/K_2_HPO_4_ (0.1/0.1 M), 100 °C, 30 min.

The XPS analyses were in full agreement with the XRD results. In the Auger spectrum obtained following the addition of NaOH (Figure 6a), the peaks corresponding to the Ag_2_O-AgO mixture, observed at kinetic energies of 350.7 eV (Ag M_5_N_45_N_45_) and 356.6 eV (Ag M_4_N_45_N_45_) in Figure 4a, disappeared. This confirms that the medium can be regenerated by the addition of NaOH. The spectrum exhibited a profile similar to that observed at the start of the first reaction cycle. The formation of silver oxide species was further confirmed after the second reaction cycle, with the characteristic Auger peaks reappearing at 350.6 and 356.5 eV (Figure 6b).

These findings suggest that AgNO_3_ can be effectively reused in subsequent reactions, with only the replenishment of K_2_S_2_O_8_ needed to maintain high levels of LA conversion and MEK yield. This study represents a significant advancement toward the development of more sustainable and environmentally friendly processes for MEK production.

## 3. Materials and Methods

### 3.1. LA Decarboxylation

Batch experiments were conducted using bushing-type ACE pressure tubes. In a typical experiment, 0.035 g of levulinic acid (3 mmol) was dissolved in 15 mL of a K_2_HPO_4_/KH_2_PO_4_ buffer solution (0.1/0.1 M in Milli-Q water, pH 6.9), followed by the addition of silver salt (3 mmol) and potassium persulfate (3 mmol). The reaction mixture was stirred and heated at 100 °C for 30 or 60 min.

The products in the liquid phase, including levulinic acid, acetic acid, and methyl ethyl ketone (MEK), were identified by GC-MS (Agilent 6980/5973, Les Ulis, France) using an Agilent J&W DB-5MS column (30 m × 0.25 mm, 0.25 µm). The injector and detector were maintained at 250 °C and 230 °C, respectively. The oven temperature was initially set to 70 °C and increased to 160 °C at a rate of 2 °C/min, followed by a further increase to 280 °C at 4 °C/min. A split ratio of 50 mL/min was used. The scan range was set from 25 to 300 m/z, and compound identification was performed using the NIST98.1 library. Levulinic acid, acetic acid, and MEK were also quantified by ^1^H NMR, with spectra recorded at 300 MHz in D_2_O using a Bruker Fourier 300 spectrometer (magnet system: 300 MHz/54 mm).

### 3.2. XPS Analyses

XPS measurements were conducted using a Thermo Scientific K-alpha instrument (Waltham, MA, USA). Photoelectron emission spectra were recorded using Al-Kα radiation (hν = 1486.6 eV) from a monochromatized source, with an X-ray spot size of 400 µm. The pass energy was set to 40 eV for high-resolution scans (and 150 eV for survey scans). A flood gun was employed to mitigate charging effects. The spectrometer was calibrated using the Au 4f_7_/_2_ (83.9 ± 0.1 eV) and Cu 2p_3_/_2_ (932.8 ± 0.1 eV) reference lines. XPS spectra were recorded in direct N(Ec) mode, with background subtraction performed using the Shirley method. Atomic concentrations were determined with an accuracy of ±10% using Scofield’s atomic sensitivity factors, accounting for the transmission function of the analyzer, which was calibrated at different pass energies using Ag 3d and Ag MNN peaks from a reference silver sample. The binding energy scale was referenced to the C 1s peak of adventitious carbon (284.7 ± 0.1 eV), and photoelectron peaks were fitted using Lorentzian/Gaussian (L/G = 30) peak fitting.

### 3.3. XRD Analyses

X-ray diffraction (XRD) patterns were obtained using a Bruker D8 Advance diffractometer equipped with a LynXeye 1D detector. The X-ray tube had a copper anode and operated without a monochromator. The primary wavelengths utilized were Kα (0.154059 nm) and Kα2 (0.15444 nm) radiation.

### 3.4. Thermodynamic Calculations

Vapor–liquid and liquid–liquid equilibrium data were evaluated using the nonrandom two-liquid (NRTL) model, implemented through Simulis Thermodynamics, a thermodynamic properties and phase equilibria calculator provided by ProSim^®^ software (https://www.prosim.net/wp-content/uploads/2019/12/Brochure-EN-Simulis-Thermodynamics_compressed.pdf, accessed on 3 October 2024) [70].

## 4. Conclusions

The decarboxylation of levulinic acid (LA) to produce methyl ethyl ketone (MEK) using the AgNO_3_/K_2_S_2_O_8_ system was systematically studied. MEK production was observed even in the absence of AgNO_3_, but the presence of K_2_S_2_O_8_ was essential for the reaction, even when Ag(II) species were involved. These results confirm that sulfate radicals (SO_4_•⁻) are directly responsible for LA decarboxylation, supporting a radical-based mechanism for MEK synthesis.

XRD and XPS analyses of the solid phases and surfaces throughout the reaction revealed that Ag(I) species were predominant, with minor traces of Ag(II) (as AgO) and elemental Ag(0), without any apparent reaction limitation. Acidification of the reaction medium was found to inhibit LA decarboxylation. By adjusting the pH to 5 and adding one more equivalent of K_2_S_2_O_8_ after 30 min of reaction, a high LA conversion (up to 99%) and MEK yield of 87% were achieved in a second reaction cycle. Furthermore, AgNO_3_ recycling was demonstrated to be feasible, making this process a promising step toward continuous MEK production, with K_2_S_2_O_8_ being the only required reagent for replenishment.

## Figures and Tables

**Table 1:** Influence of AgNO_3_ and K_2_S_2_O_8_ on LA decarboxylation.

#	n_eq_ * AgNO_3_/K_2_S_2_O_8_	Initial pH	Final pH	LA Conversion (%)	AcOH Yield (%)	MEK Yield (%)
1	1/0	5	4	0.0	0.0	0.0
2	0/0.25	5	5	3.6	1.0	2.6
3	0/0.5	5	5	12.1	3.3	8.8
4	0/1	4	2	13.6	6.8	6.8
5	0/2	4	1	18.8	14.5	4.3

* n_eq_ = number of equivalents with respect to LA. Experimental conditions: K_2_HPO_4_/KH_2_PO_4_ (0.1/0.1 M), 100 °C, 30 min.

**Table 2:** Effect of different Ag salts on LA decarboxylation.

#	Ag Salt	n_eq_ Ag Salt/K_2_S_2_O_8_	Initial pH	Final pH	LA Conversion (%)	AcOH Yield (%)	MEK Yield (%)
1	AgNO_3_	1/1	5	2	46.9	14.4	32.5
2	AgCl	1/1	6	5	38.7	13.5	25.2
3	Ag_2_O	1/1	4	3	54.3	17.8	36.5
4	AgO	1/1	5	3	57.6	22.9	34.8
5	AgO	1/0	4	1	0.0	0.0	0.0

Experimental conditions: K_2_HPO_4_/KH_2_PO_4_ (0.1/0.1 M), 100 °C, 30 min. Entry #1 is used for comparison in the following tables.

**Table 3:** Effect of AgNO_3_/K_2_S_2_O_8_ ratio on LA decarboxylation.

#	n_eq_ AgNO_3_/K_2_S_2_O_8_	Initial pH	Final pH	LA Conversion (%)	AcOH Yield (%)	MEK Yield (%)
1	0.5/0.5	5	3	22.8	13.6	9.2
2	1/0.5	5	2	34.2	15.8	18.4
3	1/1	5	2	46.9	14.4	32.5
4	1/2	4	1	49.2	21.3	27.9

Experimental conditions: K_2_HPO_4_/KH_2_PO_4_ (0.1/0.1 M), 100 °C, 30 min.

**Table 4:** Effect on LA decarboxylation of aqueous composition and initial pH.

#	Aqueous Composition	Solution Concentration [M]	Solution pH *	Initial pH	Final pH	LA Conversion (%)	AcOH Yield (%)	MEK Yield (%)
1	Water	-	6	3	1	29.6	14.8	14.8
2	KCl/NaOH	0.2	12	4	1	48.8	12.3	36.5
3	K_2_HPO_4_/KH_2_PO_4_	0.2	7	5	2	46.9	14.4	32.5
4	K_2_HPO_4_/KH_2_PO_4_	0.2	8	5	2	47.6	15.7	31.0
5	Na_2_HPO_4_/NaH_2_PO_4_	0.2	7	4	2	50.5	10.9	39.6
6	KH_2_PO_4_	0.2	4	3	1	48.9	21.3	27.9
7	K_2_HPO_4_	0.2	8	5	1	50.0	11.0	39.0
8	NaOH	9 × 10^–4^	8	5	1	30.5	9.7	20.8

* Solution pH measured at 25 °C before the addition of reactants. Experimental conditions: AgNO_3_/K_2_S_2_O_8_ (1/1), 100 °C, 30 min.

**Table 5:** Improvement of LA conversion and MEK yield following the adjustment of reaction pH.

#	Description of Reaction	n_eq_ AgNO_3_/K_2_S_2_O_8_	Initial pH	Final pH	LA Conversion (%)	AcOH Yield (%)	MEK Yield (%)
1	Initial reaction: medium 1 (M1) ^a^	1/1	5	2	46.9	14.4	32.5
	**After adjustment at pH 5**						
2	Addition of NaOH to M1 ^b^	0/0	5	2	43.8	11.8	32.0
3	Addition of NaOH/K_2_S_2_O_8_ to M1 ^b^	0/1	5	1	97.9	11.3	86.6
	**Without adjustment of pH**						
4	Addition of K_2_S_2_O_8_ to M1 ^b^	0/1	2	1	54.3	22.8	31.5
5	Addition of AgNO_3_/K_2_S_2_O_8_ to M1 ^b^	1/1	2	1	67.0	28.7	38.3

Experimental conditions: AgNO_3_/K_2_S_2_O_8_ (1/1), KH_2_PO_4_/K_2_HPO_4_ (0.1/0.1 M), 100 °C. ^a^ The first cycle of 30 min, ^b^ after the second cycle of 30 min.

## Data Availability

Data can be found in the Ph.D. thesis cited below.
